# Supplementary material for: Impact of Surface Chemistry of Silicon Nanoparticles on the Structural and Electrochemical Properties of Si/Ni3.4Sn4 Composite Anode for Li-Ion Batteries
Source: Nanomaterials (Basel). 2020 Dec 24;11(1):18. doi: 10.3390/nano11010018 (PMC7823592; doi:10.3390/nano11010018)
Supplement: Supplementary file 1 [file nanomaterials-11-00018-s001.pdf]

Impact of surface chemistry of silicon nanoparticles on the structural and electrochemical properties of Si/$Ni_{3.4}Sn_4$/Al composite anode for Li-ion batteries.

Tahar Azib[a], Claire Thaury[a,b], Fermin Cuevas[a,*], Eric Leroy[a], Christian Jordy[b], Nicolas Marx[c] and Michel Latroche[a]

[a] *Univ Paris Est Creteil, CNRS, ICMPE, UMR 7182, 2 rue Henri Dunant, 94320 Thiais, France*

[b] *SAFT Batteries, 113 Bd. Alfred Daney, 33074 Bordeaux, France*

[c] *Umicore, Watertorenstraat 33, 2250 Olen, Belgium*

Supplementary information

Full microstructural characterization for the three Si powders used as precursors of the composite synthesis is given in Figure S1 (Si_R, bare Si), Figure S2 (Si_C, carbon-coated Si) and Figure S3 (Si_O, oxide-coated Si).

Figure S4 shows the evolution of the XRD patterns as a function of milling time for the Si_R-NiSn (top), Si_C-NiSn (middle) and Si_O-NiSn (bottom) composites. Their structural properties at the end of milling (20 h) were analyzed by the Rietveld and Loopstra method. Graphical output is shown in Figure S5. Figure S6 shows a High-Resolution TEM image of the SiC -NiSn composite showing the crystallite size of silicon and its $Ni_{3.4}Sn_4$ counterparts.

* Corresponding author: cuevas@icmpe.cnrs.fr;

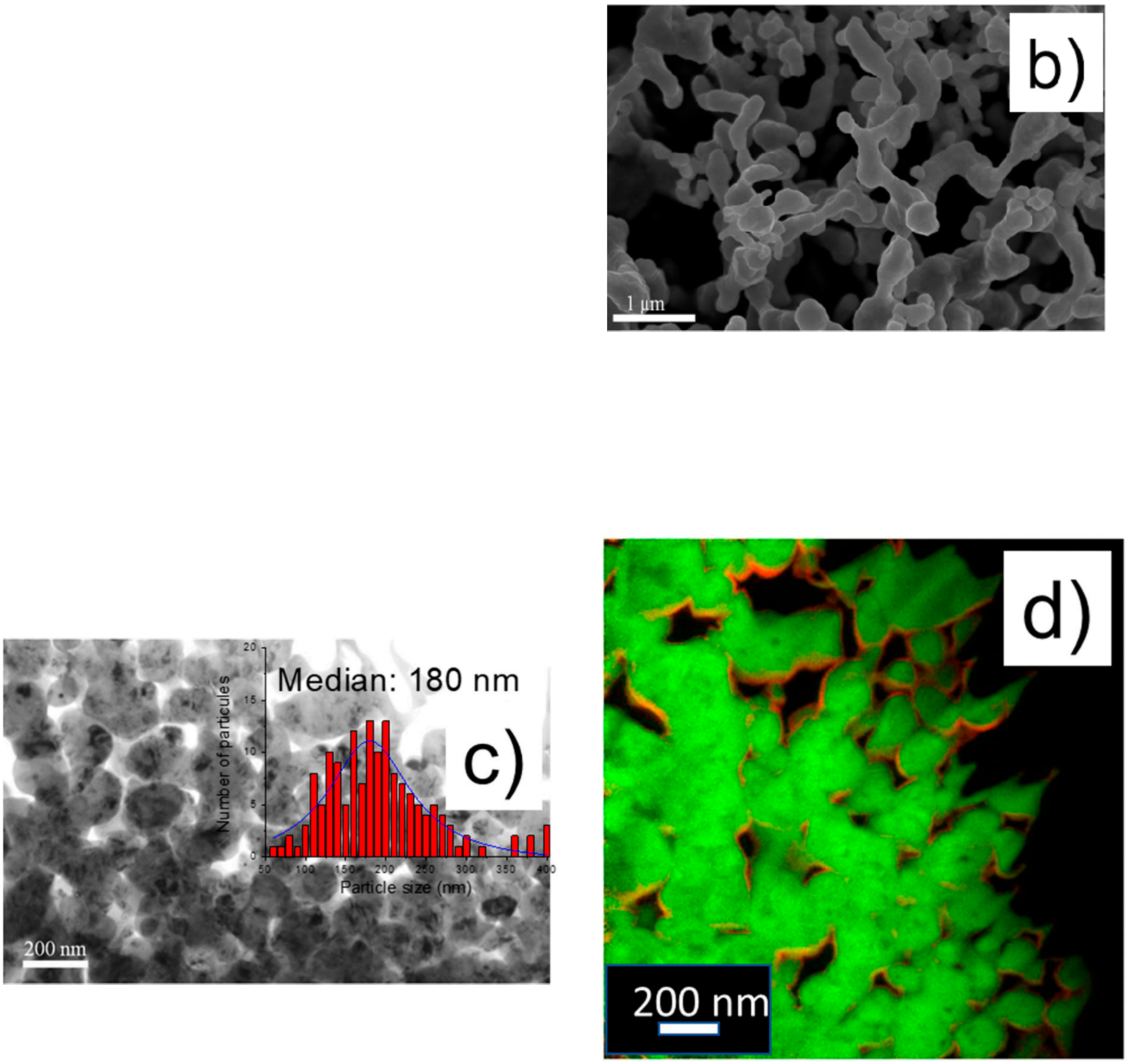

Fig. S1 : microstructural characterization of bare Si powder Si_R, a) Rietveld analysis of the XRD pattern with peak indexation to Si phase (S.G Fd-3m), b) SEM images in secondary electron mode, c) TEM image in bright field and particle size distribution analysis, d) EFTEM elemental mapping (silicon in green, oxygen in red).

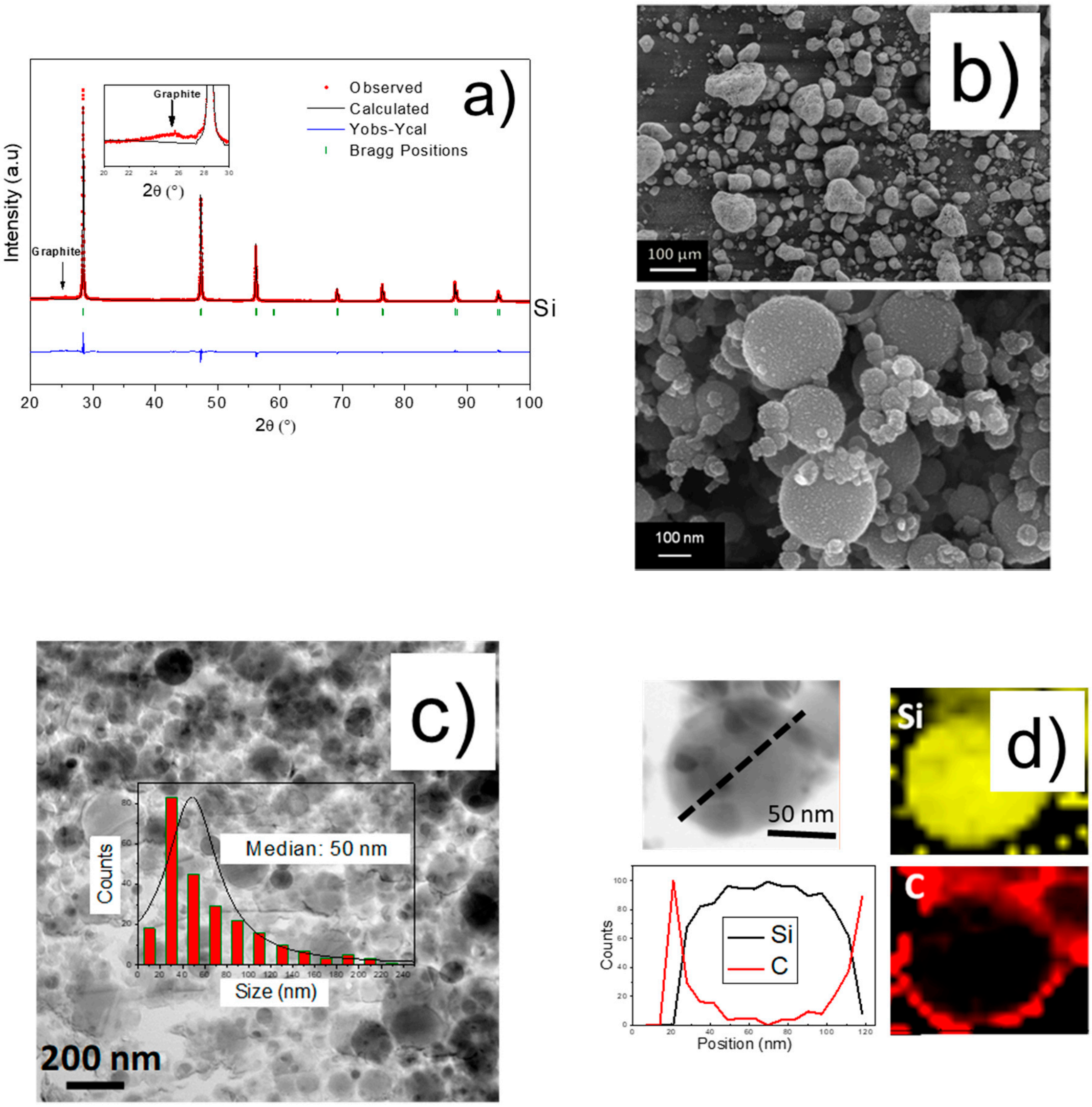

Fig. S2 : microstructural characterization of carbon-coated Si powder Si$_C$, a) Rietveld analysis of the XRD pattern with Bragg positions of silicon; the arrow indicates the broad peak around 25°-2θ shown in the inset and attributed to graphite, b) SEM images in secondary electron mode, c) TEM image in bright field and particle size distribution analysis, d) EDX-STEM elemental mapping (silicon in yellow, carbon in red) line-scan analysis (silicon in black, oxygen in red)

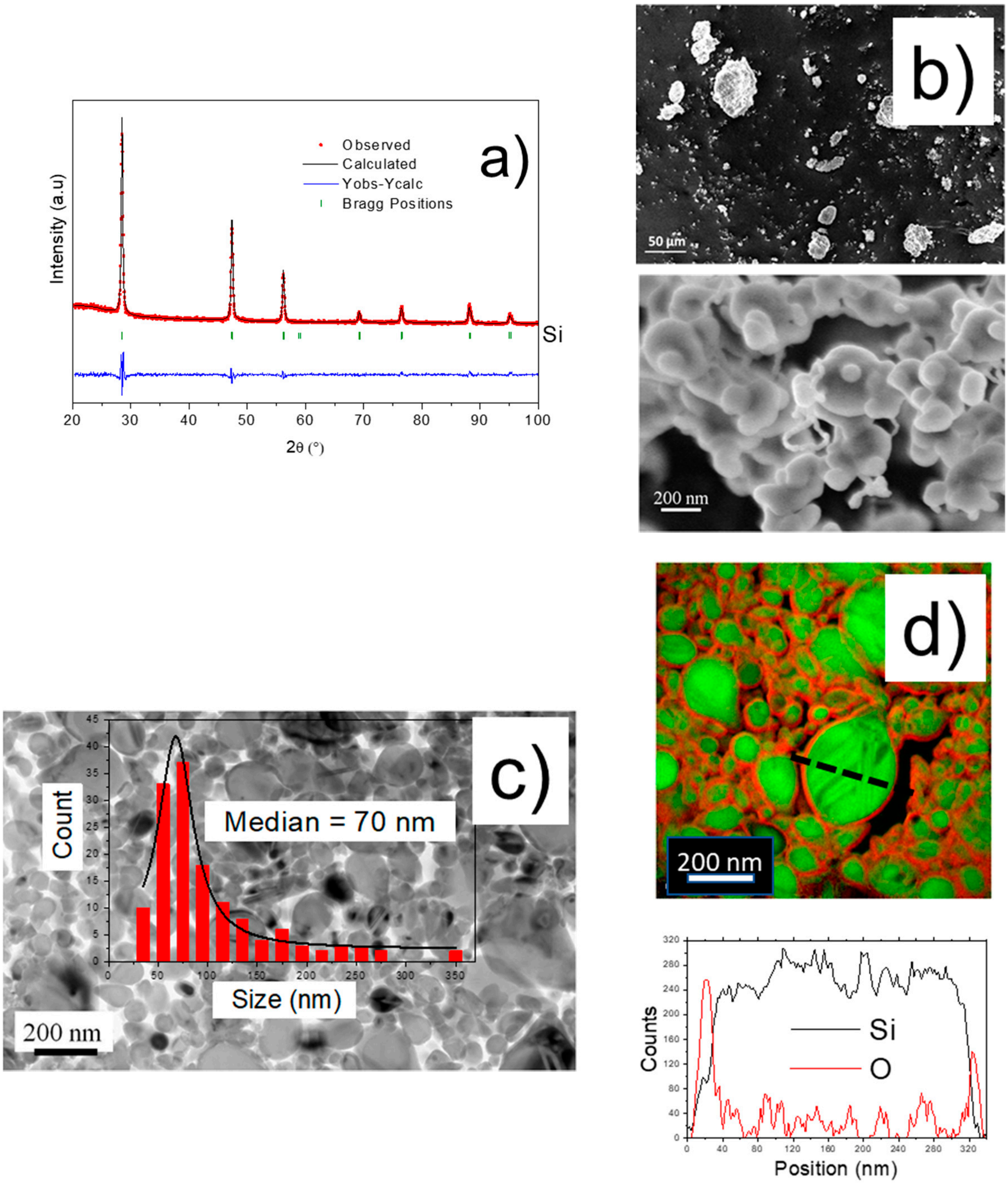

Fig. S3 : microstructural characterization of oxide-coated Si powder Si$_C$, a) Rietveld analysis of the XRD pattern with Bragg positions of silicon, b) SEM images in secondary electron mode, c) TEM image in bright field and particle size distribution analysis, d) EFTEM elemental mapping (silicon in green, oxygen in red) and line-scan analysis (silicon in black, oxygen in red)

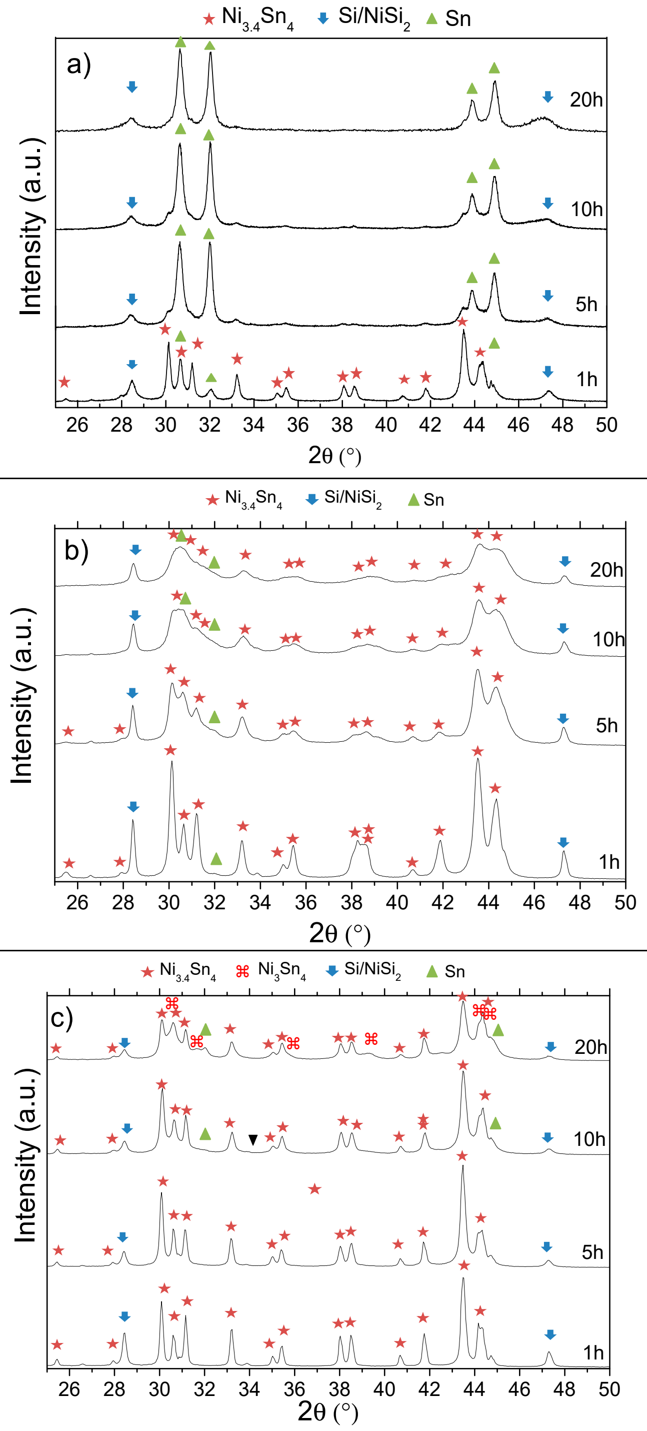

Fig. S4 :Evolution of the XRD patterns as a function of milling time (1 to 20h) for the Si_R-NiSn (a), Si_C-NiSn (b) and Si_O-NiSn (c) composites. Peak assignments are shown on top of each figure.

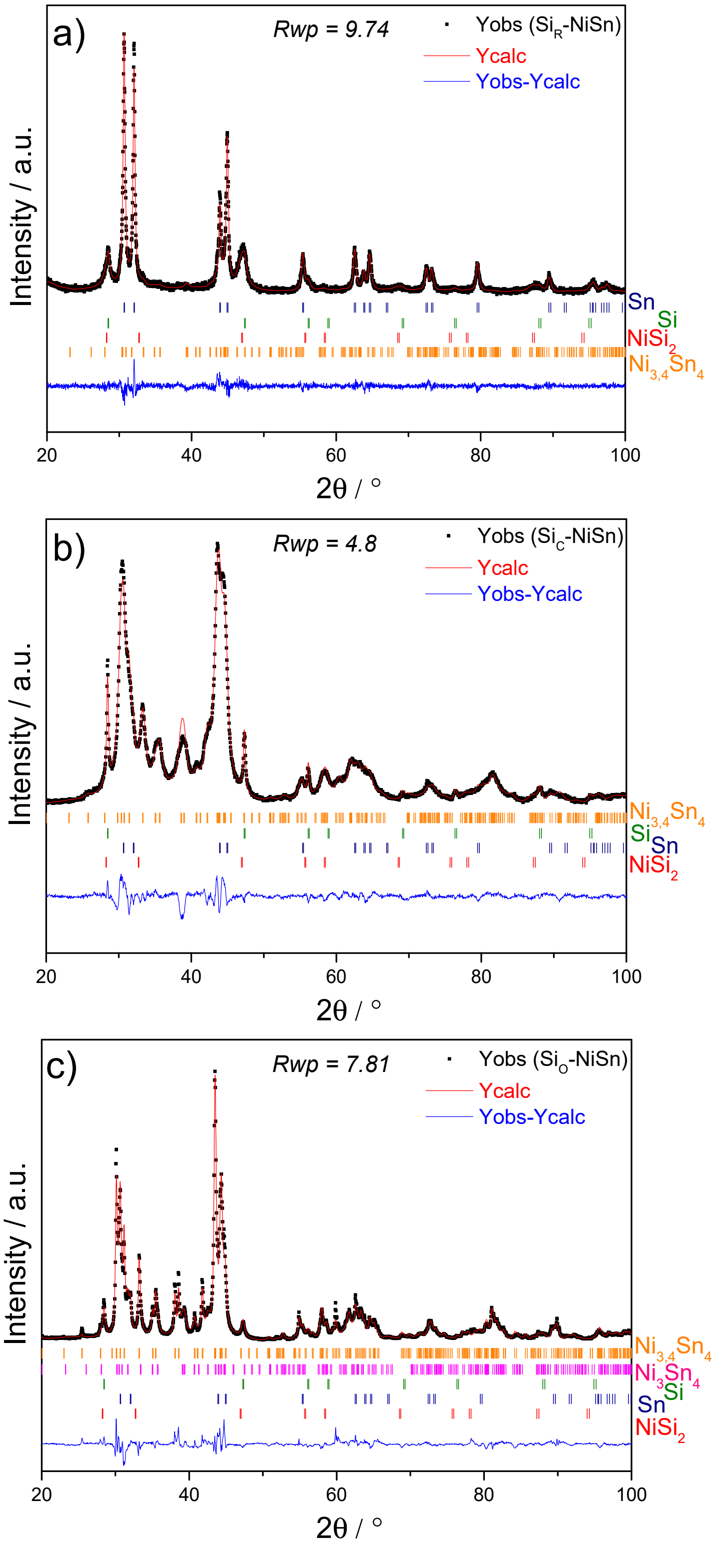

Fig. S5 : Graphical output of Rietveld analysis for Si_R-NiSn (top), Si_C-NiSn (middle) and Si_O-NiSn (bottom) composites.

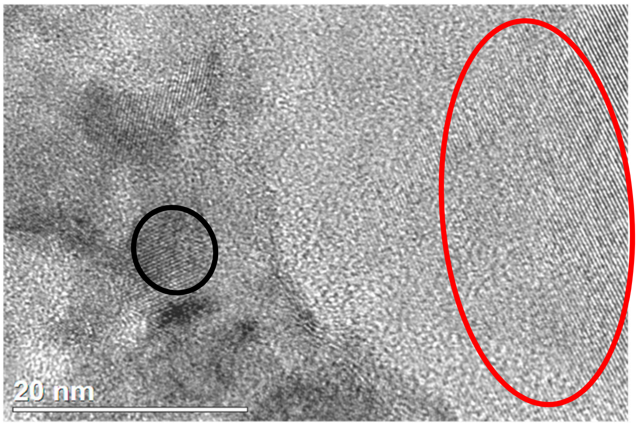

Figure S6. High-resolution TEM image of the Si_C -NiSn composite showing the typical coherent size of silicon (red area) and $Ni_{3.4}Sn_4$ (black area) crystallites.
